# Photolon Nanoporous Photoactive Material with Antibacterial Activity and Label-Free Noncontact Method for Free Radical Detection

**DOI:** 10.3390/ijms23010279

**Published:** 2021-12-28

**Authors:** Igor Buzalewicz, Iwona Hołowacz, Anna K. Matczuk, Mateusz Guźniczak, Dominika Skrzela, Magdalena Karwańska, Alina Wieliczko, Katarzyna Kowal, Agnieszka Ulatowska-Jarża

**Affiliations:** 1 Department of Biomedical Engineering, Faculty of Fundamental Problems of Technology, Wrocław University of Science and Technology, 27 Wybrzeże S. Wyspiańskiego St., 50-370 Wroclaw, Poland; iwona.holowacz@pwr.edu.pl (I.H.); 249986@student.pwr.edu.pl (M.G.); 251114@student.pwr.edu.pl (D.S.); agnieszka.ulatowska-jarza@pwr.edu.pl (A.U.-J.); 2 Department of Pathology, Division of Microbiology, Faculty of Veterinary Medicine, Wrocław University of Environmental and Life Sciences, 31 C.K. Norwida St., 51-375 Wroclaw, Poland; anna.matczuk@upwr.edu.pl; 3 Department of Epizootiology and Veterinary Administration with Clinic of Infectious Diseases, Wrocław University of Environmental and Life Sciences, 45 Grunwaldzki Square, 50-366 Wroclaw, Poland; magdalena.karwanska@upwr.edu.pl (M.K.); alina.wieliczko@upwr.edu.pl (A.W.); 4 Independent Researcher, 54-104 Wroclaw, Poland; katarzyna.m.kowal@gmail.com

**Keywords:** photodynamic effect, Photolon, bacteria photoinactivation, photoactive materials, free radicals detection, Kelvin probe microscopy, digital holographic tomography, confocal microscopy

## Abstract

The worldwide increase in bacterial resistance and healthcare-associated bacterial infections pose a serious threat to human health. The antimicrobial photodynamic method reveals the opportunity for a new therapeutic approach that is based on the limited delivery of photosensitizer from the material surface. Nanoporous inorganic–organic composites were obtained by entrapment of photosensitizer Photolon in polysiloxanes that was prepared by the sol–gel method. The material was characterized by its porosity, optical properties (fluorescence and absorbance), and laser-induced antimicrobial activity against *Staphylococcus epidermidis*, *Staphylococcus aureus*, *Pseudomonas aeruginosa*, and *Escherichia coli*. The permanent encapsulation of Photolon in the silica coating and the antimicrobial efficiency was confirmed by confocal microscope and digital holotomography. The generation of free radicals from nanoporous surfaces was proved by scanning Kelvin probe microscopy. For the first time, it was confirmed that Kelvin probe microscopy can be a label-free, noncontact alternative to other conventional methods based on fluorescence or chemiluminescence probes, etc. It was confirmed that the proposed photoactive coating enables the antibacterial photodynamic effect based on free radicals released from the surface of the coating. The highest bactericidal efficiency of the proposed coating was 87.16%. This coating can selectively limit the multiplication of bacterial cells, while protecting the environment and reducing the risk of surface contamination.

## 1. Introduction

Every day humans are constantly exposed to the simplest cellular organisms in our world—bacteria. However, despite the simplicity of the cellular structure, bacteria have an important impact on the functioning and state of our health e.g., beneficial bacterial flora that influence our immunological system. The pathogenic bacteria strains that are responsible for many infectious diseases such as sepsis, pneumonia, abscess, meningitis, gastroenteritis, and food poisoning, require rapid cures which target the invading bacterial species. According to the observed increase in the resistance of bacteria to commonly-used antibiotics around the world, the World Health Organization (WHO) announced in 2017 the list of priority pathogens with the highest need for new antibiotics [1]. Furthermore, the WHO warns that more than 16 million patients per year die from healthcare-associated infections (HCAI) (including bacterial infection). Recently, the COVID-19 pandemic, that was caused by the severe acute respiratory syndrome virus (SARS-CoV-2), has shown that secondary bacterial infections of the respiratory tract are of great concern for patients that are overcoming the disease [2]. The bacteria species that are indicated as the most COVID-19 bacterial co-infections include: *Haemophilus influenzae*, *Staphylococcus aureus, Klebsiella pneumoniae*, *Mycoplasma pneumoniae*, *Streptococcus pneumoniae*, *Pseudomonas aeruginosa*, and *Escherichia coli* [3,4]. Therefore, it is necessary to develop new antibiotics, antibacterial therapies, or agents to combat the antibiotic resistance of bacteria worldwide.

**Figure 1:**
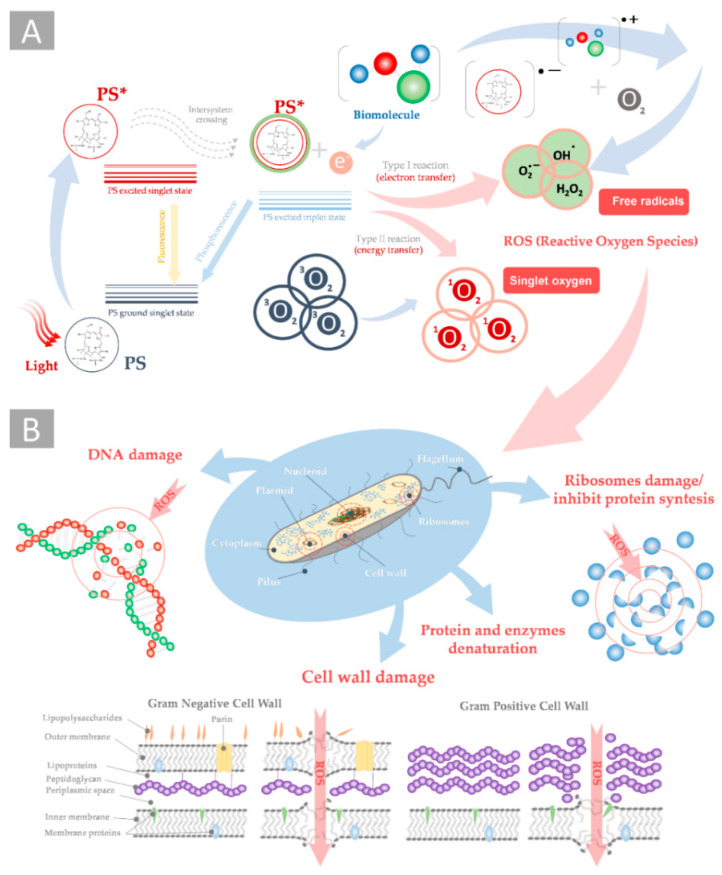
Schema of antimicrobial photodynamic therapy: (**A**) photoexcitation and generation of ROS by type I and II photodynamic reactions and (**B**) the mechanism of the ROS-induced destruction of bacteria cell structures leading to bacteria cell death (*—indicates the photoexcitation).

Antimicrobial photodynamic therapy (aPDT) emerged as an effective solution against resistant strains. Generally, photodynamic therapy is a type of photochemotherapy that is used to treat cancer, but it can also be used for certain non-cancerous treatments against the overgrowth of unwanted or abnormal cells, as well as foreign cells (bacteria, fungi, and viruses) [5,6]. The aPDT procedure requires the exposure of target bacteria cells to a non-toxic dye, termed a photosensitizer (PS), followed by irradiation with visible light of an appropriate wavelength in the presence of sufficient amounts of molecular oxygen (see Figure 1A).

PS can produce reactive oxygen species (ROS) via type I reactions (involving the generation of free radicals) or type II reactions (involving the generation of excited singlet oxygen). Effective treatment with aPDT requires the accumulation of PS in the immediate vicinity of the cell: it can be located in the cell’s neighborhood, accumulate in the bacteria cell walls or membranes, or can penetrate the cell interior. There are several mechanisms that lead to the killing of bacterial cells by ROS generation after PS photoexcitation (see Figure 1B).

In our recent in vitro studies, the properties of the novel antibacterial PS-doped photoactive silica coating and its bactericidal efficiency were examined. The silica matrix that was used with tetraethol orthosilicate to ethyl alcohol molar ratio 20 was previously used by our group for the construction of medical sensors [7], for the production of the applicators for laser-induced interstitial therapy [8], and for intravascular photodynamic therapy as a stent coating [9]. Here, we propose another approach towards the direct delivery of free radicals from the surface of the photoactive coating. Photolon (Ph) PS encapsulated inside the pores of a silica matrix enables the generation of free radicals not only from the surface of the coating but also from an interconnected small pores network of the deposited coating still containing the entrapped single PS molecules in the silica matrix to single living bacterial cells. It was reported that the appropriate porosity of the silica matrix forces the monomolecular packing of PS in a layer, which leads the increase in fluorescence intensity and an enhanced photodynamic effect. Therefore, in this paper, seven different silica matrices with encapsulated Ph PS were examined by mercury porosimetry and spectrophotometric measurements to select the coating with the best photoactive properties.

To verify the generation of free radicals after PS photoexcitation, scanning Kelvin probe microscopy (KPM) was used. It is a non-contact capacitively-coupled voltage measurement technique that detects the contact potential difference (U_CPD_) or surface potential (SP) as the potential differences between the probe conductive tip and the analyzed material surface directly beneath the probe. The U_CPD_ sensing is based on the dissimilarity in the work function (WF) of the probe tip metal and the examined material and it is thus very sensitive to any surface contamination or changes in its chemical composition [10]. Modern KPM or scanning surface potential microscopy (SSPM) also find applications in the biological examination of tissues [11], single cells [12] (including bacteria cells [13]), or biomaterials [14]. In our study, the KPM was used to confirm the presence and generation rate of free radicals by the proposed photoactive coating. Our approach assumed that this reaction, based on the electron/proton transfer between the PS that is encapsulated in the pores of the silica matrix and biomolecules from the surrounding medium and generate the specific electric charge distribution on the photoactive coating, correlated with the changes of the U_CPD_. To our knowledge, this is the first attempt to use KPM for the detection of free radical generation. The positive results confirm that KPM can be a significant fully label-free alternative for conventional techniques [15] that are usually applied for the detection of free radicals and ROS that is based on fluorescence probes [16], chemiluminescence probes [17], chromatography [18], electro-spin resonance probes [19], or fluorescence proteins [20].

The proposed photoactive coating permanently bonds the PS molecules on the surface of the silica matrix or pores. Therefore, to validate the potential release of PS from the surface, an additional fluorescence confocal microscopic examination was performed in the presence of the bacteria cells. If the PS was released from the silica surface, it would penetrate the bacterial cells, which could be tracked by the presence of the luminescence signal inside the cell. These results were also confirmed by digital holographic tomography (DHT) [21,22] a label-free quantitative phase imaging technique, as our previous studies confirmed that the penetration of single bacteria cells by PS is related to the increase in the average refractive index (RI) of the cell [23]. The performed examinations confirmed that, in case of the lack of PS accumulation inside the bacteria cells, their death is caused directly by the generated free radicals.

Finally, the assessment of the aPDT efficiency of the proposed photoactive coating was evaluated based on the examination of the four bacteria species (2 g positive, 2 g negative): *Staphylococcus epidermidis*, *Staphylococcus aureus*, *Pseudomonas aeruginosa*, and *Escherichia coli* were cultivated on the surface of the proposed photoactive coatings performed by DHT.

The results confirmed that the proposed PS-doped photoactive coating can be used for the light-induced generation of free radicals from the coating surface and offer a high aPDT efficiency of 35.59% for *S. aureus* to 87.16% for *E. coli*. The proposed coating enables the delivery of free radicals directly to single living bacteria cells prior to the formation of mature biofilms from the coating that are deposited on a glass or metal substrate. The coating can be applied both as a protective layer and as an antibacterial layer on medical devices or surfaces. Furthermore, it was verified that KPM can be used for the detection of the free radicals’ generation on the coating surface, which, in comparison to the other conventional techniques, enables the detection of free radicals in a truly label-free, nondestructive, and non-contact manner.

## 2. Results and Discussions

### 2.1. Pore Radius Distribution in Examined Coatings

**Figure 2:**
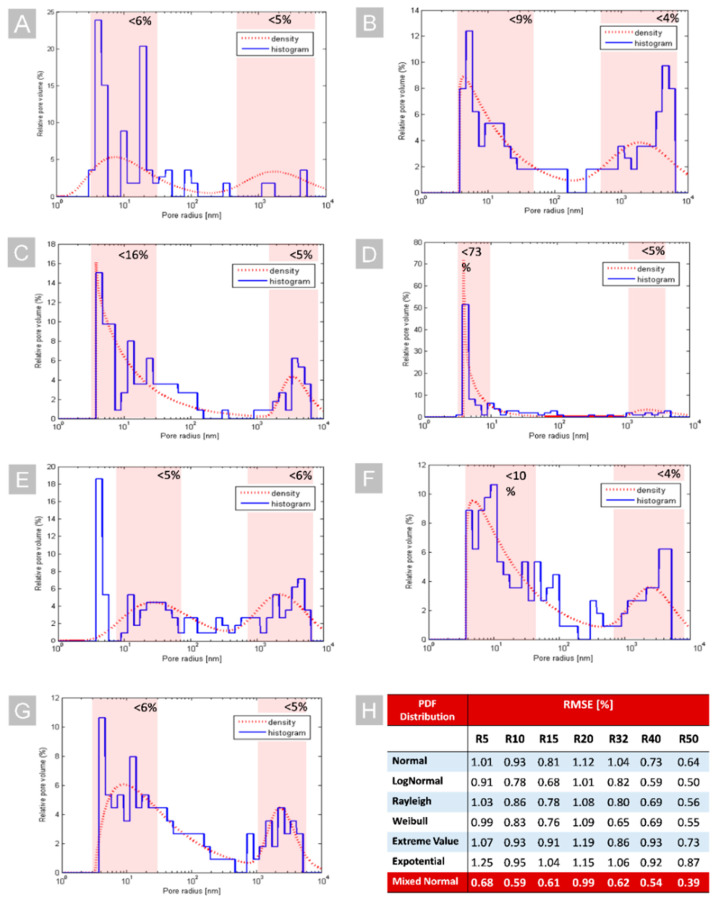
The pore radius distribution (histograms-solid blue line, PDF fitting-dashed red line) of different materials: R 5 (**A**), R 10 (**B**), R 15 (**C**), R 20 (**D**), R 32 (**E**), R 40 (**F**), R 50 (**G**), and the comparison of the goodness of the PDF fitting that was based on the used RMSE (**H**). The red regions represent the different fractions of pores with two predominant radius ranges.

The experimental results that showed the distribution of pores versus the relative pore volume in a sample of non-doped coatings R 5, 10, 15, 20, 32, 40, and 50 materials are depicted in Figure 2. Histograms of the pores’ radius distribution were fitted by seven different distributions to estimate the best probability density functions (PDFs). The lowest RSME (Root mean square error) was obtained for the mixed normal distribution (see Figure 2H) and was used for further analysis. Based on the estimated PDFs, it can be seen that, in the case of most of the coatings (see Figure 2A–G) excluding R 20, they have a bimodal distribution of the radius of the pores. In each case, two fractions of pores with different radiuses (<100 nm and >100 nm) could be distinguished. These fractions of pores represented 4 to 16% of the relative volume of the pores. However, a different situation was observed in the case of the R20 material, since the percentage of the relative pore volume for the pores with the smallest radius was nearly seven times higher than in the case of other materials. In this case, nearly 73% of the relative pore volume corresponded to pores with a radius that was smaller than 10 nm (see Figure 2D). Therefore, it can be stated that the R 20 material exhibited a monomodal distribution of the pore radius with a predominant radius of single nanometers. Such porosity of the R20 silica matrix forces the monomolecular packing of PS in the layer, which causes the increase in fluorescence intensity and the enhanced photodynamic effect, which was proved previously [24].

The proposed approach, that is based on the packing of the PS monomer, allows it to be retained in the silica matrix which enables more precise control of its concentration. Although, following the path of the antitumor application of PS in combination with mesoporous silica nanoparticle carriers (MSN) [25,26], MSN-PS conjugate against *C. albicans* was also designed [27], but such solutions are based on the PS delivery directly to the cells. However, such an approach requires taking into account the different pathways of anionic and cationic photosensitizers into the bacteria [28]. Therefore, the delivery of universal PS inside the cell is difficult due to the different structures of Gram-negative and Gram-positive, as well as the effect of the efflux pump. On the other hand, our approach that is based on PS that was permanently encapsulated in a silica matrix is more universal, since the death of bacteria is not obtained by ROS or the internal generation of free radicals inside the cell, but by the external generation of free radicals from the surface leading to destruction of the cell walls and membranes, whether cationic or anionic.

### 2.2. Spectrophotometric Examination of Photoactive Coating

**Figure 3:**
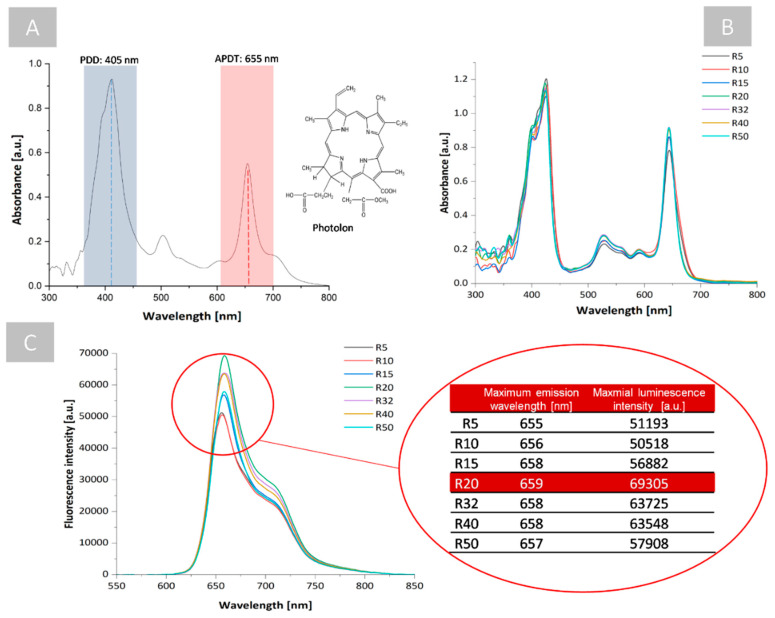
Exemplary results of the spectrophotometric measurements: (**A**) Ph absorption in ethyl alcohol. (**B**) Ph absorption and (**C**) luminescence in different silica coatings (excitation wavelength: 405 nm) with a table demonstrating the variation of the luminescence intensity.

The absorbance and luminescence of all the materials that were doped with Ph as a PS were analyzed. The absorption spectrum of Ph shows a strong band at 405 nm (Soret band) and weaker Q-bands at 500, 595, and 655 nm (see Figure 3A). The Soret band at 405 nm is generally used for photoexcitation and the generation of PS luminescence, while the Q band at 655 nm can be used for the generation of free radicals. The results that were obtained have shown that the changes that were related to the type of material that was examined (R 5-R 50) generally affected the absorbance of PS in the Q bands, particularly at 655 nm, which was responsible for the performance of antimicrobial photodynamic therapy (see Figure 3B). In the case of the R 20 material, the highest increase in absorbance was observed in this band. However, the significant differences that were related to the type of the examined materials were also observed in the luminescence spectra (see Figure 3C).

Upon excitation at 405 nm, the emission spectrum of Ph exhibited a strong maximum from 655 to 659 nm for all colloid with the highest luminescence intensity for the R 20 material, which was related to the high solubility of Ph and the structural changes in the silica colloid that lead to small pores formation in the solid material. The predominant number of small pores in the material (single nanometers) favored the monomeric form of PS, preventing PS self-aggregation; self-aggregated states can reduce fluorescence quantum yields, triplet states, and ROS/free radical generation, thereby reducing photoactivity [29]. However, this process, in some cases, can improve the generation of radical species, for example hydroxyl and peroxyl radicals, even if PS aggregation reduces the generation of ^1^O_2_. [30]. Nevertheless, most of the aggregated PSs are less photochemically active due to the non-radiative deactivation of the excited states, which shortens the fluorescence lifetime [31]. The R 20 material has a monomodal distribution of pores which was chosen due to the possibility of keeping PS in a non-aggregated form that is manifested by a high intensity of luminescence (see Figure 3C). This effect enables the easy prevention from PS self-aggregation regardless of the kind of PS that is used or without the need for additional modifications of the PS focused on obtaining the weak intermolecular interactions or steric effect preventing self-aggregation [32,33]. Moreover, it should be pointed out that in the biological media, the interactions of PS with biomolecules or with membranes may alter the monomer/aggregate equilibrium. Our approach limits these problems since it forces the monomeric form of PS by its encapsulation in the nano-pores.

### 2.3. Kelvin Probe Microscopic Characterization of the Photoactive Coatings and Free Radicals Generation

In the case of the phototoxicity of Photolon, ROS generation via a type I mechanism was reported [34]. The use of type I free radical scavengers in light-activated Ph was shown to cause a significant reduction in ROS, whereas the use of singlet oxygen scavengers or an enhancer of singlet oxygen production had no significant effect. Another study showed that the type of ROS that is produced after Photolon-PDT is H_2_O_2_, but the authors could not rule out the possibility of the involvement of other free radical species, so this mechanism has not yet been addressed [35]. These studies indicate that the phototoxicity of Ph is related to the type I reaction leading to the proton or electron transfer between the photoexcited PS and that the surrounding matter can affect the surface distribution of the electric charge. Therefore, in a recent study, Kelvin probe microscopy (KPM) was used to characterize the conditions of free radical generation resulting from the photoexcitation of the PS inside the examined porous silica materials. The photoexcitation of Ph that is accumulated inside pores enables the local generation of free radicals that are necessary for photodynamic treatment on the coating surface, which should lead to changes in U_CPD_.

**Figure 4:**
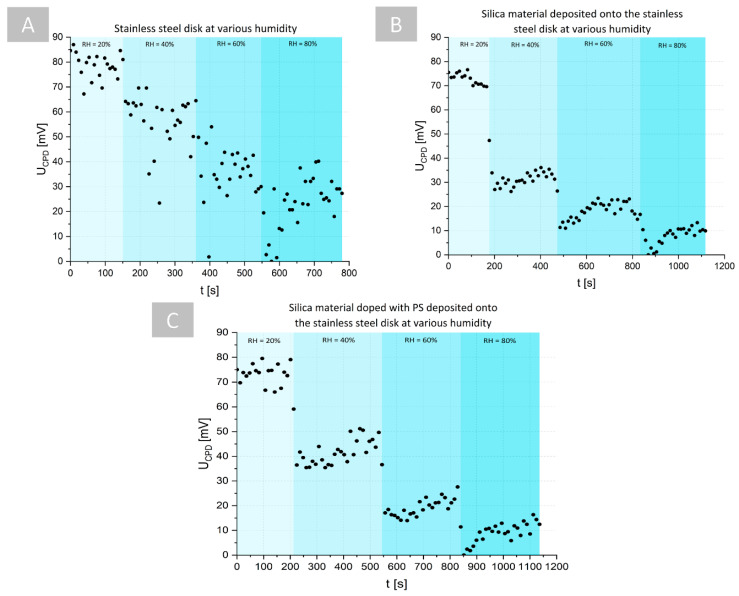
The influence of the relative humidity (RH) on the time-resolved U_CPD_ measurements: (**A**) pure stainless steel, (**B**) silica material deposited on stainless steel, and (**C**) photoactive silica material deposited on stainless steel.

To verify this assumption, in the first stage of the proposed examination, the time-resolved influence of the relative humidity (RH) conditions on the U_CPD_ measurements were analyzed. In the case of stainless steel (see Figure 4A) and silica material that was deposited on the surface of stainless steel (see Figure 4B), a decrease in U_CPD_ was observed with an increase in RH. However, in the case of the silica material, the time-resolved changes of the U_CPD_ for specific RH exhibited significantly lower variations than for only stainless steel, suggesting a higher stabilization of the measurement conditions on the surface of the silica material. The same trend was observed in the case of the silica material that was doped with PS that was deposited on stainless steel (see Figure 4C). This can be explained by the decrease in effect of the U_CPD_ decrease for high RH values that are reported in [36], where the influence of RH on U_CPD_ was analyzed in the case of individual few-layer graphene films that were deposited on a SiO_2_/Si wafer. It was proven that at high humidity, there is a high diffusion of charges on the coating surface, and no charge can be accumulated on the surface that is composed of adsorbed water film on the silica coating, which leads to surface discharging and a decrease of the U_CPD_. It should be also pointed out that, in the case of the silica coating with/without PS deposited on stainless steel, the ranges of the U_CPD_ values that changed were slightly lower than in the case of the stainless steel only without any coating, which suggests that the deposition of the coating on the stainless steel decreases the U_CPD_.

**Figure 5:**
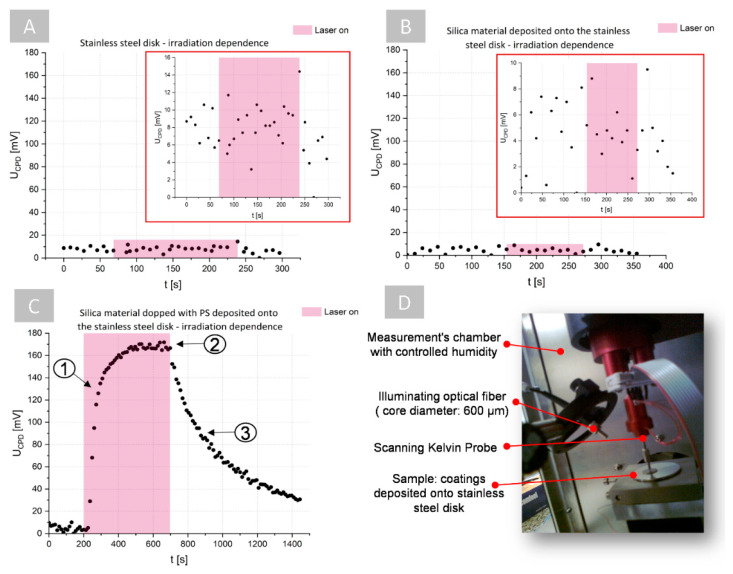
The changes of U_CPD_ of different surfaces (RH = 80%): (**A**) stainless steel, (**B**) stainless steel with silica coating, and (**C**) stainless steel with PS-doped silica coating depending on the laser irradiation at 655 nm wavelength (red regions represent surface exposition to laser light—laser on, 1—generation of free radicals, 2—saturation of the generation of free radicals, and 3—exponential decrease in free radical generation), (**D**) the used measurement setup.

The antimicrobial photodynamic treatment requires the PS photoexcitation and the generation of reactive oxygen species (free radicals: superoxide anion radical O_2_^•−^, a hydroxyl radical OH^•^, or hydrogen peroxide H_2_O_2_ and singlet oxygen ^1^O_2_) by a type I or II photodynamic reaction. However, only the type I reaction leads to electrical charging of the coating surface due to the electron/proton transfer between the surrounding medium and the PS, which can be resolved by KPM measurements. Therefore, it was necessary to perform the examination of the influence of the PS-doped silica coating photoexcitation and generation of the free radicals via the type I reaction on the U_CPD_. In the measurement humidity chamber of the KPM system, the additional mounted optical fiber that was coupled with the laser source was used for sample illumination (see Figure 5D).

The time-resolved U_CPD_ measurements were performed on different surfaces (stainless steel, stainless steel with silica coating, and stainless steel with a PS-doped silica coating) that were exposed under illumination at a wavelength of 655 nm. During irradiation, for the stainless steel (see Figure 5A) and stainless steel with silica coating (see Figure 5B) there were no significant differences in U_CPD_ values. Moreover, the variation of the U_CPD_ values before, during, and after irradiation was smaller in the case of the surface with a silica coating, which could be associated with the already discussed above effect of the decrease in U_CPD_ after deposition of the coating on the surface of the stainless steel. On the other hand, quite a different dependence was observed in the case of the irradiation of the PS-doped silica coating that was deposited on stainless steel (see Figure 5C). After the start of laser exposure, the U_CPD_ significantly increased with time, so that at the end of the exposure it had a value 16-times higher than the initial value before illumination. After exposure (laser off), the U_CPD_ exhibits exponential decays over time. These results suggest that U_CPD_ was related to the changes of the electric charge distribution on the examined surface and reveals the proton and electron transfer between the PS and the surrounding matter caused by PS-photoexcitation and the type I reaction. During light exposure, proton/electron transfer and free radical generation initially increased (see 1 in Figure 5C) until saturation states that were related to the quantum efficiency of PS and its concentration in the coating were achieved (see 2 in Figure 5C). After exposure, free radical generation is stopped and PS relaxation occurs (3 in Figure 5C), which is related to the exponential decrease in U_CPD_. These particular stages of PS photoexcitation, the generation of free radicals, the achievement of the maximum quantum efficiency of PS, and its relaxation are evidently observable in the changes in U_CPD_ over time. Therefore, time-dependent U_CPD_ measurements enables distinguishing of the three main stages of the PS photoexcitation (see Figure 5C): (1) the generation of the free radical (the high increase of the free radical amount), (2) quantum efficiency saturation (nearly constant amount of free radical), and (3) relaxation (the exponential decay of the free radical’s amount).

**Figure 6:**
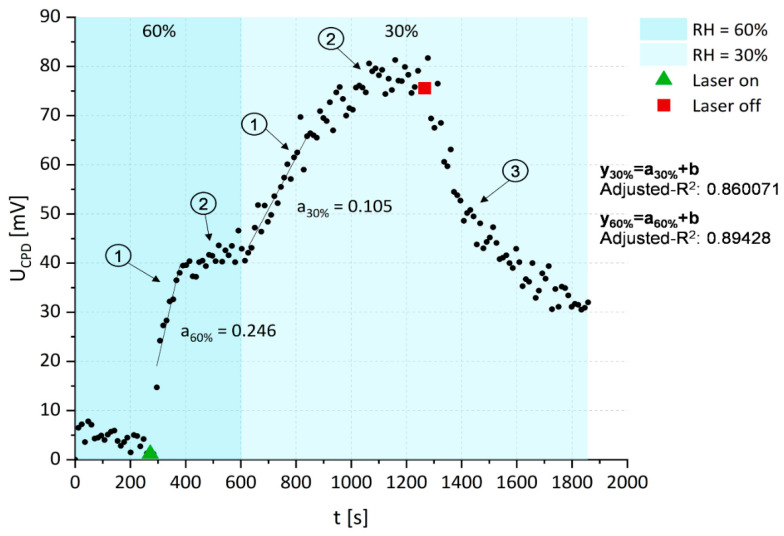
The changes of U_CPD_ on stainless steel with a PS-doped silica coating depending on the laser irradiation at 655 nm wavelength and the relative humidity in the measurement chamber (1—high rate of the free radicals generation, 2—saturation of free radical generation, and 3—decay of the free radicals generation).

The last stage of this study was to examine the influence of two different humidity conditions (RH = 60%, RH = 30%) and the different content of water that was related to the surface on the U_CPD_ measurement of free radical’s generation of photoexcited PS-doped silica coatings in stainless steel (see Figure 6).

It was shown that, as in previous cases, the exposure of the photoactive coating on the laser irradiation led to a high increase in the U_CPD_, which was related to the proton/electron transfer characteristics for the type I photodynamic reaction, which increases until the saturation of the quantum efficiency is achieved and the amount of free radicals will grow. However, based on the time-dependent changes that were obtained from U_CPD_, it was shown that the dynamic rate of free radical generation (stage 1 in Figure 6) and the efficiency of this process depended on the RH (the water content in the medium surrounding the coating). Based on the linear regression coefficients that were fitted to the slope of the U_CPD_ changes over time under light exposure, it can be seen that the rate of free radical generation was significantly higher in the environment with the higher content of water. This suggests that the water can affect the amount of free radicals that are generated or the quantum efficiency of the PS as it provides the molecules that enable the transfer of protons and electrons between them and the photosensitizer that is necessary for the generation of free radicals according to the type I reaction.

Alternatively, this effect can be associated with the already discussed discharging of the surface at high humidity. The water film adsorption on the surface of the PS-doped silica coating may be responsible for the lower potential difference that was measured between the scanning probe and the sample surface for RH = 60%, what limited the range of U_CPD_ values changes that were correlated with the amount of the generated free radicals. As a consequence, the limitation of the RH can improve the sensitivity of the U_CPD_ measurement on the change of the free radical amount. However, despite this effect, the obtained results confirmed that the generation of free radicals via the type I reaction after the photoexcitation of the Ph-doped silica coatings was correlated with the changes of U_CPD_ that were measured by KPM in a label-free manner. Therefore, the KPM measurements can be an alternative for the conventionally-used methods for the examination of free radical generation and the rate of this process.

### 2.4. Examination of the Antimicrobial Activity of Photoactive Coating

#### 2.4.1. The Examination of the Possible Ph Accumulation inside the Bacteria Cells by Confocal Microscopy and DHT Subsection

**Figure 7:**
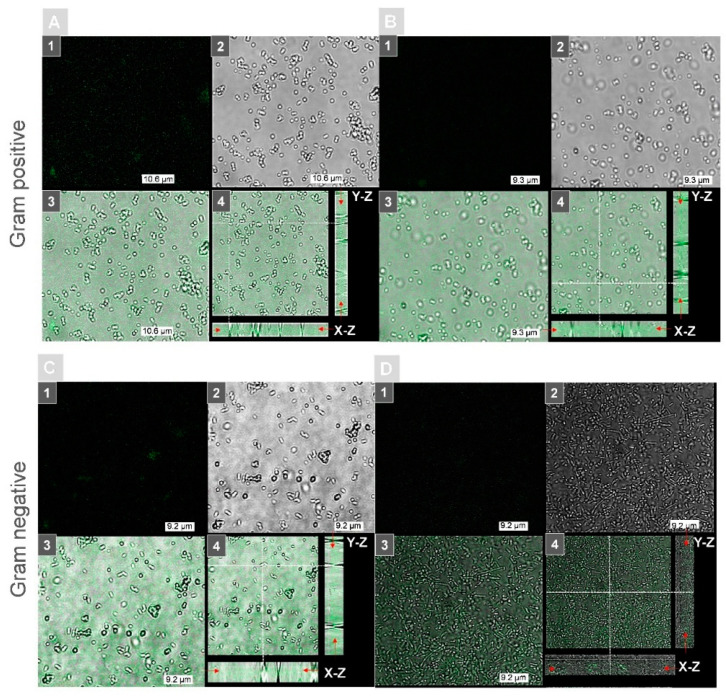
Examination of the Ph localization end encapsulation inside the R 20 silica coating that was performed by fluorescence confocal microscopy for (**A**) *S. aureus*, (**B**) *S. epidermidis*, (**C**) *P. aeruginosa*, and (**D**) *E. coli* bacteria cells that were cultivated on Ph-doped photoactive coatings: (1) fluorescence image (excitation wavelength: 405 nm), (2) DIC image, (3) combined fluorescence-DIC images, and (4) the combined fluorescence-DIC images with axial cross sections: X-Z/Y-Z (the dashed white lines indicate the cross section through a single cell).

The main aim of the study was to obtain a photoactive surface with PS that was encapsulated in a porous silica matrix and to perform a photodynamic bacteria inactivation that was based on free radicals that were released from the surface after a laser-induced type I photodynamic reaction. Therefore, first, the allocation of Ph that was encapsulated in silica matrix carriers was studied using fluorescence confocal microscopy after incubation of the bacteria cells on the surface of the Ph-doped R 20 silica matrix. The exemplary results are shown in Figure 7. As one can see, a Ph fluorescence was observed in all the samples of bacteria species. Moreover, the fluorescence was present only in the background region that was not occupied by single cells, which confirmed that Ph was present only in the silica coatings. On the basis of the axial cross sections (XZ/YZ) across the single cells, it was shown that the lack of fluorescent presence in the region that was occupied by bacteria cells excludes the possible accumulation of Ph on the cell wall or penetration of the cell interior. Moreover, it is possible to determine the direct location of the Ph-doped coating surface on the basis of the highest fluorescence signal on the cross-sections of the combined fluorescence-DIC (indicated by the red arrow on Figure 7A–D4), which was located out of the bacteria cells. These results confirmed that Ph is permanently encapsulated inside the pores of the silica matrix and that the potential bactericidal effect can only be achieved by the free radicals that are released on the surface of the proposed photoactive coating.

**Figure 8:**
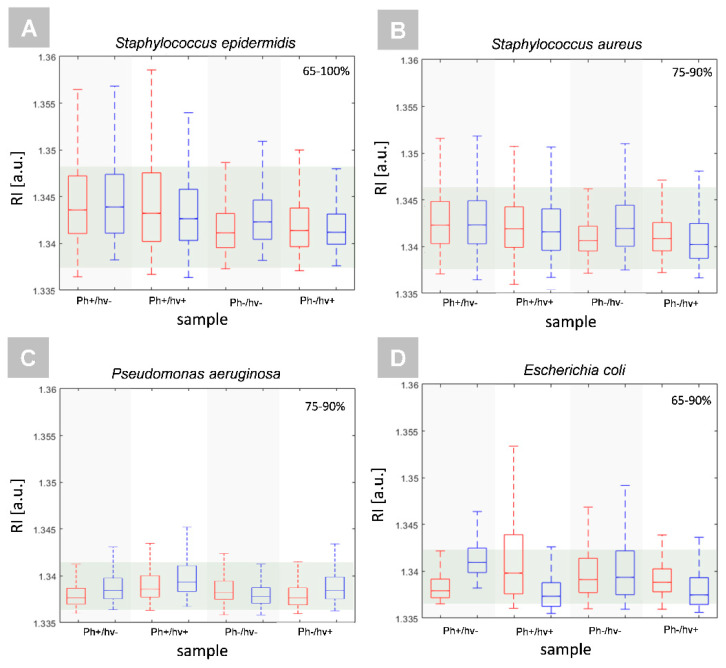
Representative box plots of RI values of single cells of Gram positive: (**A**) *S. epidermidis* and (**B**) *S. aureus*, and Gram negative bacteria species: (**C**) *P. aeruginosa* and (**D**) *E. coli* that were cultivated on different surfaces (Ph+/hν-: PS-doped silica coating not illuminated, Ph+/hν+: PS-doped silica coating illuminated, Ph-/hν- silica coating not illuminated, and Ph-/hν+: silica coating illuminated). The red boxes represent the results before PS photoexcitation, and the blue boxes 24 h after PS photoexcitation (aPDT treatment). The percentage values determine the percentage of observations with similar RI values as indicated by the green region.

The same observations were confirmed by digital holographic tomography (DHT), enabling the reconstruction of the 3D refractive index distribution. Our previous results have shown that quantitative analysis of the 3D-RI distributions of single bacteria cells revealed the increase in the RI values that were related to PS accumulation within the cellular structures of bacteria [23]. Therefore, the same approach was used in this study to verify if Ph penetrates the bacterial cells. The examination was performed before the illumination of the bacterial cells on surfaces with photoactive coatings and 24 h after illumination—photodynamic treatment. The results that were obtained from the averaged 3D-RI values of single bacteria cells that were grown on different surfaces, illuminated, and not illuminated are shown on Figure 8A–D. The red boxes represent the distribution of the RI values before illumination and the blue boxes are 24 h after illumination.

On each box, the central mark indicates the median, and the bottom and top edges of the box indicate the 25th and 75th percentiles, respectively. The whiskers extend to the most extreme data points that are not considered as outliers. Therefore, it can be seen that in the case of all samples and bacteria species, similar averaged RI values of single cells represented at least from 65% to 90% of all the observations, which confirms the lack of differences between the samples and that the PS was not penetrating the cells. However, it should be noted that the increase in the average RI of single cells that were related to the increase in cell density can be associated not only with the accumulation of PS in the cell interior or cell membranes, but also with physiological processes such as cell division, which was confirmed by our previous results [23].

#### 2.4.2. The Examination of Antibacterial Efficiency of Photoactive Coatings by DHT

**Figure 9:**
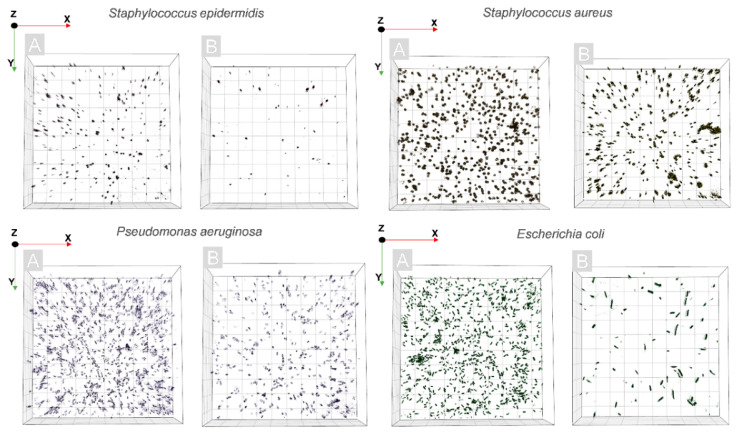
Representative digitally stained (based on the RI values) cells of *S. epidermidis, S. aureus, P. aeruginosa, E. coli* on the surface of the Ph-doped silica coating before (**A**) and 24 h after photoexcitation (**B**).

To determine the efficiency of the antibacterial photodynamic treatment after photoexcitation of the Ph-doped silica coatings and the generation of free radicals, the 3D-RI distributions of bacterial cells that were reconstructed by DHT were used. On the basis of the RI values of single cells, it is possible to digitally stain in truly label-free manner. The exemplary visualization of the digitally stained cells on the surface of the examined coatings are shown in Figure 9. The efficiency of antibacterial photodynamic therapy is based on the killing of single-bacteria cells by the laser-induced release of free radicals from the photoactive coating after photoexcitation, and it is directly related to the number of cells on the examined surface before and after photoexcitation. Based on the reconstructed 3D-RI tomograms of single cells by DHT and the proposed algorithm for processing 3D-RI tomograms, it was possible to determine the number of cells on the surface of each analyzed coating.

**Figure 10:**
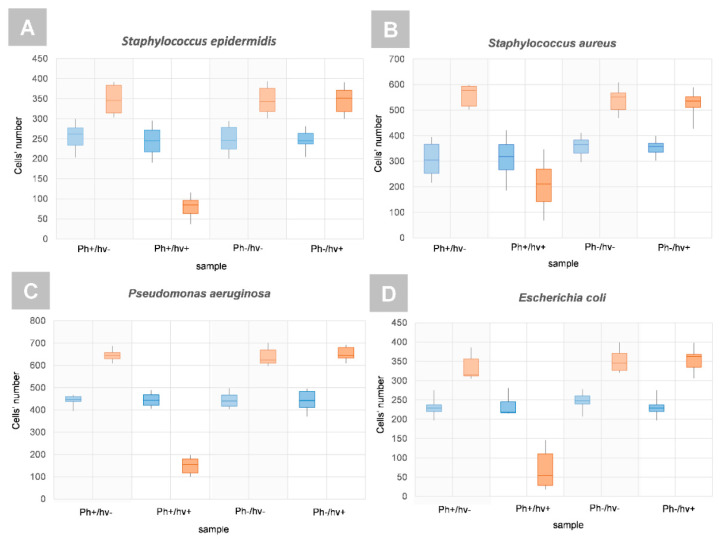
Representative boxplots of the number of single cells of Gram-positive: (**A**) *S. epidermidis* and (**B**) *S. aureus* and Gram-negative bacteria species: (**C**) *P. aeruginosa* and (**D**) *E. coli* that were cultivated on different surfaces (Ph+/hν−: PS-doped silica coating not illuminated, Ph+/hν+: PS-doped silica coating illuminated, Ph−/hν-: silica coating not illuminated, and Ph−/hν+: silica coating illuminated). The red boxes represent the results before PS-photoexcitation and the blue boxes 24 h after PS-photoexcitation (aPDT treatment).

The results of the cell number distributions are shown in Figure 10. It was shown that the number of number of limitations of the bacterial cells number could be observed only in the case of the PS-doped silica coating for all the examined bacteria species, both Gram-positive and Gram-negative. Based on the mean number N of cells that was determined on the Ph-doped silica coating before (NPh+/hv+) and 24 h after illumination (NPh+/hv+24 h), it was possible to evaluate the aPDT efficiency that was expressed as (NPh+/hv+—NPh+/hv+ 24 h) / NPh+/hv+) for all the analyzed bacteria species: 65.31% for *S. epidermidis*, 35.59% for *S. aureus*, 65.12% for *P. aeruginosa*, and 87.16% for *E. coli*. These results confirm that the proposed Ph-doped silica coatings are capable of performing efficient antibacterial photodynamic treatment based on the generation of free radicals that are released from the surface of the coating. Moreover, the proposed photoactive coating can be used to combat Gram-negative and Gram-positive bacteria species.

Recently, it was shown that it is possible to obtain a higher inactivation of *E. coli* and *S. aureus* by porphyrin derivatives even up to 7 logs [37]. However, it should be noted that, in our case, a 17 nM concentration of PS was used which was significantly lower than 20 µM in the reported case. Therefore, increasing the PS concentration can improve bactericidal efficiency. Furthermore, our results are better than in some other alternatives such as [38] in which the bactericidal efficiency that was achieved by aPDT was equal to nearly 20% and 25% in the case of *S. aureus* and *E. coli*, respectively. Therefore, it can be stated that our results provide the foundation for further studies that are aimed at the development of the antimicrobial photodynamic inactivation technique. Moreover, it should be pointed out that in both references that are cited, the aPDT was based on the PS accumulated inside the bacterial cells and *E. coli* had a weaker response to photodynamic inactivation. Our approach that was based on the release of free radicals from the surface of the coating allows shortening of the antimicrobial process as it bypasses the step of accumulation of PS inside the bacterial cells and consideration of the blockage of the efflux pump. Furthermore, the results that were obtained demonstrate that the bactericidal effect on *E. coli* in our approach is significantly better, suggesting that the external generation of free radicals from the surface of the coating can be more effective than internal generation from the PS that is accumulated within the bacteria cell.

## 3. Materials and Methods

### 3.1. Antimicrobial Photoactive Coatings Preparation

The silica materials were prepared from colloid precursor 99% tetraethol orthosilicate (brand Aldrich, Merck Life Science, Poznań, Poland), and 96% ethyl alcohol (Avantor Performance Materials Poland S.A., Gliwice, Poland) in solvent to precursor molar ratio R5, R10, R15, R20, R32, R40, and R50. The mixture was stirred for 4 h with a speed of 400 rot/min at room temperature. The base materials were prepared from 100 µL of silica colloid that was deposited in glass bottom micro-dishes (µ-Dish 35 mm low, Ibidi GmbH, Gräfelfing, Germany) or stainless steel SS316L discs (diameter 10 mm, thickness 0.3 mm) by the spin coating method (speed 3000 rpm for 30 s). Photolon (18-carboxy-20-(carboxymethyl)-8-ethenyl-13-ethyl-2,3-dihydro-3,3,12,17-tetramethyl-21H,23H-porphin-2-propionicacid) (Belmedpreparaty, Minsk, Belarus) was used as photosensitizer (PS). The antibacterial coatings were prepared from the base silica materials that were doped with PS solutions to obtain the 17 nmol of PS in each micro-dish. The micro-dishes and SS discs were temporally attached to the spin-coater table using an anti-slip pad with gecko pattern. This solution let us to avoid the glue residues that are present in optical or electrical signals when double-sided tape is used. All µ-dishes and stainless steel discs with coatings were stored overnight in sterile Petri dishes in darkness at a temperature that was equal to 4 °C.

### 3.2. Examination of Pores Size Distribution in Materials and Data Analysis

The evaluation of the pore size distribution and pore morphology was carried out from data that were obtained by mercury porosimetry (Pascal 440 mercury porosimeter, Thermo Fisher Scientific, Waltham, MA, USA). The raw data that were produced by Pascal 440 was used to create histograms representing the pore radius distribution vs. the relative pore volume. Then, for each histogram, the probability density function (PDF) was fitted in MATLAB^®^ software (version R2020a, MathWorks, Natick, MA, USA). A total of seven different PDF distributions were used for this purpose: normal (Gaussian), lognormal, Rayleigh, Weibull, extreme value, exponential, and mixed normal (mixture Gaussian) to limit the possible fitting errors. For estimation of the goodness of fit, the RMSE (root mean squared error) measure was used. The RMSE is a measure of the differences between the observation values as predicted by a fitting model and the observed values that were applied. This parameter estimates the standard deviation of the random component in the data. When RMSE takes values closer to 0, it indicates that the fit will be more useful for predicting the observation values.

### 3.3. Spectrophotometric Measurements

To examine the absorption/emission spectra of the examined PS and to determine the photoexcitation conditions, spectrophotometric measurements were performed with an AVA-Spec 3648 spectrophotometer (Avantes Inc., Apeldoorn, The Netherlands) with 2 nm spectral resolution. The Avalight-DH-S-BAL deuterium halogen lamp (Avantes Inc., Apeldoorn, The Netherlands) working in the wavelength range 200–1100 nm was used as the light source. As an excitation source in the measurement of PS luminescence measurement, the continuous wave semiconductor laser λ = 405 nm (TOPGaN, Warsaw, Poland) was applied.

### 3.4. Kelvin Probe Microscopy Examination

One of the objectives of a recent study was to verify the KPM as a label-free alternative for the detection of free radical generation by PS. It is a noninvasive vibrating capacitor technique that is used to measure changes in work function or for nonmetal surface potential between a conducting tip and a conducting (or partially conducting) specimen. KPM is a scanning probe technique that is capable of measuring the local distribution of contact potential difference (U_CPD_), which is a measure of the electrical surface potential or the work function of the sample. The KPM signal is carried out by applying a voltage to a conducting probe. Scanning the probe across the structure of interest produces a map of the electrostatic interaction at each point of the sample. Stopping the probe tip in one place gives information about time-dependent processes. Kelvin probe microscopy (KP Technology, Wick, UK) with the Kelvin probe measurement system version DELTA 5+ ver. The 5.05 software was used to demonstrate the presence of a local surface potential for PS-doped materials resulting from spin coating deposition. A stainless steel probe tip with a diameter of 0.1 mm was used. The probe tip was cleaned with absolute ethyl alcohol and air-dried prior to operation. A medical and food grade stainless steel (grade 316L, UNS S31603) was selected as the model metallic substrate. A total of 60 round samples that were 10 mm in diameter were machined directly from flat metal of 0.3 mm thickness and were polished both sides by waterproof silicon carbide abrasive sheets that were described as 1200, 2000, and 2500 grit. The samples were then ultrasonically cleaned in distilled water, acetone, absolute ethanol (both analytical grade), and distilled water again (15 min in each bath); naturally dried; and stored in clean laboratory air to naturally re-passivate their surface. A total of twenty polished, uncoated disks were stored in a desiccator, 20 were coated with silica material, and last 20 were coated with Ph-doped silica as described in Section 3.1. All the KPM measurements were conducted in a humidity chamber with an RH control unit working in the range of 20–80%. The samples were irradiated via a sealed passage in the chamber wall with an optical fiber that was connected to a laser diode.

### 3.5. Bacterial Sample Preparation

A total of four bacteria species (2 Gram positive, 2 Gram negative): *Staphylococcus epidermidis* (Polish Collection of Microorganisms: PCM 2532), *Staphylococcus aureus* (ATCC 25923), *Pseudomonas aeruginosa* (ATCC 27853), and *Escherichia coli* (ATCC 25922) that were capable of biofilm formation were examined. The cultures were obtained from the Department of Epizootiology and Veterinary Administration with the Clinic of Infectious Diseases of the Wroclaw University of Environmental and Life Sciences. A single colony of each species was inoculated in Brain Heart Infusion (BHI) medium at 37 °C. The overnight culture stocks were measured with the MacFarland scale estimation; 0.5 McF was taken, which is approximately 1.5 × 10^8^ bacterial cells per ml. The bacteria suspensions were then diluted in NaCl solution and approximately 1.5 × 10^5^ bacterial cells were applied to the dish for attachment. The diluted bacterial suspension was then aliquoted using 1 mL per well of µ-dish. For each bacteria species, the bacteria suspension was applied on two types of silica coatings: without Photolon photosensitizer (Ph-) and photoactive coating with PS (Ph+) in 5 repetitions. Biofilm formation begins with irreversible cell attachment to the surface [39]. Therefore, to examine only the cells that attached to the surface of the prepared coatings after 2 h of incubation at 37 °C, the supernatant with the planktonic cells was removed and the well was washed twice with 2 mL of NaCl (0.9%), followed by the addition of 2 mL of NaCl (0.9%). This procedure for the preparation of the biofilm samples for the characterization of different antimicrobial coatings or agents has already been reported [40]. The samples were then incubated under the same conditions for the next 24 h at 37 °C. After that, the 8 samples with bacteria cells that were attached to the surface of silica coatings with/without PS were irradiated for 2 min (bright controls marked as: Ph−/hν+, Ph+/hν+) and 8 samples of the same coatings were not irradiated (dark controls marked as: Ph+/hν−, Ph+/hν−). Then, all the samples were incubated at 37 °C for the next 24 h. Subsequently, the samples were examined by DHT. The refractive index of NaCl (0.9%) was equal to 1.335 and was measured with the Abbe refractometer (NAR-2T, minimum scale: 0.001, ATAGO Co. Ltd., Tokyo, Japan) at 20 °C.

### 3.6. Photoexcitation Conditions of Photoactive Coatings

For the PS photoexcitation, the laser-induced type I photodynamic reaction, and the generation of free radicals, the irradiation setup including a fiber-coupled laser diode with a wavelength of 655 nm (FC-655nm-1W-15070826, Changchun New Industries Optoelectronics Tech. Co., Ltd., Changchun, China) with an adjustable power unit were used. The power density was equal to 420 mW/cm^2^ and the energy density was equal to 12.5 J/cm^2^. Light power measurements were performed using a highly sensitive compact fiber photodiode power sensor (S151C, Thorlabs, USA) and a power meter (PM100D, Thorlabs, Newton, NJ, USA). All the exposures were continuously monitored by temperature measurements with a thermal imaging camera (FLIR E6, FLIR Systems, Inc., Willsonville, OR, USA) to eliminate the possibility of cell overheating. Thermal imaging confirmed that there was no temperature change that was caused by the photoexcitation.

### 3.7. Confocal Laser Microscopy

Confocal microscopic investigation of the bacterial cells on the surfaces of the analyzed coatings was performed to examine the possible penetration of single cells by PS and the accumulation of PS in the cellular structures. For PS uptake, the bacteria cells were inoculated on a micro-dish that was coated with a PS-doped silica coating. After 24 h of incubation, the samples were illuminated with a wavelength that was equal to 655 nm and incubated for the next 24 h before imaging (holotomography and confocal microscopy). The cells were then washed three times with NaCl. The confocal images of single bacteria cells were collected on a Leica TCS SPE confocal laser microscope (Leica, Wetzlar, Germany) using a 63× high numerical aperture oil immersion objective. This microscope was working in the fluorescence and differential interference contrast (DIC) modes. For the excitation of the PS and generation of the fluorescence signal indicating a location of PS, a laser with the wavelength that was equal to 405 nm and operated at 20% of maximum power was used. The optimal gain setting of 1260 V was used. DIC and fluorescence images were combined and X-Z and Y-Z cross-sections were recovered to obtain 3D information indicating that there was no penetration of the bacteria cells by PS and its accumulation in the cellular structures. If the cells’ regions were not spatially overlapping the fluorescence, then the PS was not penetrating the cells and their death can be caused only by the free radicals that were released from the photoactive coating.

### 3.8. Digital Holographic Tomography and RI-Data Processing

The examination was performed using a digital holotomographic system (3D Cell Explorer, Nanolive, Ecublens, Switzerland) for all types of samples to analyze the changes in the 3D-RI distribution of single cells and their counting. This system is based on the two-arm Mach-Zehnder interferometer and enables the registration of digital holograms (DHs) resulting from the interference between the reference beam and the object beam that were scattered by samples. The series of DHs were registered at the wavelength of 520 nm (sample exposure 0.2 mW mm^−2^) using a dry microscope objective (60×, numerical aperture NA = 0.8, Nikon) at different illumination angles of the reference beam. The series of numerically reconstructed 3D-RI tomograms that were obtained from individual samples was imported from STEVE software (version 1.6.3496, Nanolive, Ecublens, Switzerland) to MATLAB^®^ software (version R2020b, MathWorks, Natick, MA, USA). To count the individual bacteria cells, a single 2D-RI tomogram was selected, representing the 3D-RI tomogram slice for which the best contrast of the examined cells was obtained. The applied algorithm performed median filtering to reduce the artifacts that were present in the selected 2D-RI tomograms. In the next step, a contour mask was created that was based on the specified polygonal region of interest. This region was matched by appropriately specifying a threshold for the RI- values, which exceeded the RI of the medium. As a result, the number of cells was obtained which corresponded to the number of reproduced contours. Next, the contour mask was fitted on the original data (unfiltered series of 2D-RI tomograms), which enables the automatic distinguishing of the regions that were occupied by single cells and to directly determine the RI-values of each cell that is present in the sample. Additionally, it was possible to digitally stain the bacterial cells based on their spatial distribution of their RI values enabling label-free characterization. DHT was used for examination of four bacterial species that were cultivated on the four kinds of coatings samples (Ph−/hν− Ph−/hν+, Ph+/hν+, and Ph+/hν−) that were examined. For all the samples, more than 489 3D-RI tomograms containing 95 slices (2D-RI tomograms) were registered. Each 3D-RI map was reconstructed with an axial resolution of 367 nm. 

## 4. Conclusions

The examinations that were performed have shown that the predominant number of small pores in the silica matrix (single nanometers) favors the monomeric form of PS. The nearly monomodal distribution of the pores, which was chosen due to the possibility of keeping PS in a nonaggregated form, is manifested by an increase of the luminescence intensity. As this material has the smallest pores and the lowest pore-volume ratio, it strongly suggests that the presence of small pores and/or low pore-volume ratios is important for the overall biological activity of the obtained materials. The KPM examination confirms the generation of free radicals through a type I reaction on the surface of the photoactive coating after photoexcitation of Ph that is encapsulated in a silica matrix. It was shown that the presence of PS contributes to an increase in the surface potential (U_CPD)_ of the deposited material if it is doped with PS by the generation of free radicals that are measured by KPM in a label-free manner. Therefore, KPM can be an alternative to conventionally used methods. Fluorescence confocal microscopic and digital holotomographic examinations confirm that there is no accumulation of Ph within the bacterial cells and the possible antibacterial photodynamic effect can be caused only by the free radicals that are released from the coating surface. Furthermore, the results of the evaluation of the efficiency of aPDT that was performed by DHT on four bacteria species (*S. epidermidis*, *S. aureus*, *P. aeruginosa*, and *E. coli*) indicate a high bactericidal efficiency from 35.59% for *S. aureus* to 87.16% for *E. coli*, suggesting that the proposed photoactive coating varied in activity against some Gram-negative and Gram-positive species.

Future work will focus on the optimum concentration of photosensitizers in the photoactive coating, since the amount of free radicals that are generated and the effectiveness of antimicrobial therapy directly depends on the PS concentration. It is necessary to obtain the highest possible concentration of photosensitizers, however, so that no aggregation of photosensitizers takes place. Self-aggregated states can reduce fluorescence quantum yields, triplet states, and ROS/free radical generation, thereby reducing photoactivity, but, in some cases, this can improve the generation of radical species, for example hydroxyl and peroxyl radicals. Therefore, it will also be valuable to examine the different types of PSs’ that are encapsulated in silica coatings, which can improve the efficiency of free radicals that are released from the surface even after PS aggregation.

## Data Availability

All data can be obtained from authors on a reasonable request.
